# Sandwich-Structured Flexible PVA/CS@MWCNTs Composite Films with High Thermal Conductivity and Excellent Electrical Insulation

**DOI:** 10.3390/polym14122512

**Published:** 2022-06-20

**Authors:** Fanghua Luo, Chen Ma, Yuhui Tang, Lintao Zhou, Youpeng Ding, Guohua Chen

**Affiliations:** College of Materials Science and Engineering, Huaqiao University, 668 Jimei Blvd, Xiamen 361000, China; fannghua@163.com (F.L.); chenma@hqu.edu.cn (C.M.); baizhuanqianhui00@163.com (Y.T.); zardbeyondzlt@gmail.com (L.Z.); dyp11158@163.com (Y.D.)

**Keywords:** polymer-based composites, sandwich structure, flexibility, high thermal conductivity, electrical insulation

## Abstract

High thermal conductivity polymer matrix composites have become an urgent need for the thermal management of modern electronic devices. However, increasing the thermal conductivity of polymer-based composites typically results in loss of lightweight, flexibility and electrical insulation. Herein, the polyvinyl alcohol (PVA)/PVA-chitosan-adsorbed multi-walled carbon nanotubes/PVA (PVA/CS@MWCNTs) composite films with a sandwich structure were designed and fabricated by a self-construction strategy inspired by the surface film formation of milk. The obtained film simultaneously possesses high thermal conductivity, electrical insulation, and excellent flexibility. In this particular structure, the uniform intermediate layer of PVA-CS@MWCNTs contributed to improving the thermal conductivity of composite films, and the PVA distributed on both sides of the sandwich structure maintains the electrical insulation of the films (superior electrical resistivity above 10^12^ Ω·cm). It has been demonstrated that the fillers could be arranged in a horizontal direction during the scraping process. Thus, the obtained composite film exhibited high in-plane thermal conductivity of 5.312 W·m^−1^·K^−1^ at fairly low MWCNTs loading of 5 wt%, which increased by about 1190% compared with pure PVA (0.412 W·m^−1^·K^−1^). This work effectively realizes the combination of high thermal conductivity and excellent electrical insulation, which could greatly expand the application of polymer-based composite films in the area of thermal management.

## 1. Introduction

With the rapid development of electronic devices for miniaturization, high integration, and multi-function, heat accumulation is increasingly prominent [1,2]. Therefore, in order to make electronic devices stable and reliable thermal conductivity of good materials is urgently needed. Recently, highly thermal conductive and electrically insulated polymeric composites show great application potential as thermal management materials because they have lightweight, durability, flexibility, corrosion-resistant, and easy processing characteristics [3,4]. Polyvinyl alcohol (PVA) has aroused wide attention due to its excellent biocompatibility, high water solubility and insulating properties [5,6,7].

However, this severely limits the applications of polymers in the thermal management of modern electronics due to their low intrinsic thermal conductivity (~0.2 W·m^−1^·K^−1^); therefore, the introduction of fillers with high thermal conductivity into polymer substrates is identified as the ideal solution [8,9,10,11,12]. To meet the requirements of electrical insulation, many efforts have been devoted to introducing insulation fillers into the polymer matrix, such as boron nitride [13], aluminum nitride [14], silicon carbide, alumina [15] and other ceramic fillers. However, their relatively low intrinsic thermal conductivity often needs a lot of fillers to significantly improve the thermal conductivity of composites, which will result in a loss of flexibility [16]. 

Due to their high thermal conductivity (2000–6000 W/m·K), carbon nanotubes (CNTs) have attracted extensive attention as candidate materials for thermal conductivity fillers [17,18,19,20,21]. However, due to the high electrical conductivity of CNTs, a small amount of addition can significantly improve the conductivity of composites, which is an inevitable obstacle to the application of electrical insulation materials in the electronic field [22,23]. Therefore, it is still a serious challenge to achieve high thermal conductivity enhancement while retaining flexibility and excellent electrical insulation [24]. In addition, good dispersibility of fillers and strong interfacial interaction between the fillers and polymer matrix are two key factors to enhance the properties of composites [25]. However, since there is no bond between carbon nanotubes and polymer, carbon nanotubes have a strong tendency to aggregate during doping into a polymer matrix [26]. Chitosan (CS) has significant hydrophilic properties due to the high proportion of amino and hydroxyl groups [27]. In addition, CS also shows biodegradability, unique biocompatibility, antibacterial activity, and good film-forming ability [28,29]. CS and PVA blends have been studied to improve mechanical properties and provide a method for producing polymeric packaging films [30,31,32]. However, fabricating the PVA/CS@MWCNTs system for thermal conductivity management through noncovalent modification of the MWCNTs has not been reported. Therefore, CS was chosen to prevent the aggregation of MWCNTs to enhance the properties of the composites in this work.

Based on the above, sandwich-structured PVA/CS@MWCNTs composite films were designed and fabricated through a self-construction strategy, which was inspired by the surface film formation of milk. PVA-CS@MWCNTs is the middle layer of PVA/CS@MWCNTs composite film, and both sides of the PVA-CS@MWCNTs are PVA. TEM was carried out to observe the cross-section structure of the composite film. The promoting effect of CS on the uniform dispersion of MWCNTs was also displayed in this study. The effects of amounts of MWCNTs on the thermal conductivity, electrical insulation, and mechanical properties of the sandwich structured PVA/CS@MWCNTs composite film were studied in detail.

## 2. Materials and Methods

### 2.1. Chemicals and Reagents

MWCNTs (diameters: 10–20 nm, purity: >98%) were provided by Chengdu Organic Chemistry Co. Ltd., Chinese Academy of Sciences, Chengdu, China. PVA (molecular weight: 130.14200, degree of polymerization: 1700 ± 50, alcoholysis degree: 88%, CP) and chitosan (molecular weight: 300000, deacetylation is 95%) were supplied by Shanghai Aladdin Biochemical Technology Co., Ltd., Shanghai, China. The molecular weight units of Cs and PVA are all 1. Glycerol and glacial acetic acid were offered by Sinopharm Chemical Reagent Co., Ltd., Shanghai, China.

### 2.2. Preparation of CS@MWCNTs

Chitosan solution with a concentration of 1.0 wt% was prepared by magnetic stirring at 75 °C for 5 h after a certain amount of chitosan was dissolved in 1.0 wt% acetic acid aqueous solution. Then, a certain amount of MWNTs was placed in 1.0 wt% chitosan aqueous solution, magnetic stirring at 75 °C for 24 h, and high-frequency ultrasound for 5 min to obtain the black suspension of chitosan-coated MWNTs. 

### 2.3. Preparation of PVA/Glycerol Solution

First, 9 g PVA particles were added to 90 mL of deionized water and stirred with magnetic force at room temperature until the solution was transparent. Then, 1 g of glycerol was added as plasticizer and stirred with magnetic force for 2 h at 95 °C. The resulting PVA/glycerol solution was cooled to room temperature, and bubbles were removed for later use.

### 2.4. Preparation of PVA/CS@MWCNTs Composite Films

The CS@MWCNTs solution (4 mg/mL) was added to the PVA/H_2_O/glycerol solution; first, it was subjected to high-frequency ultrasound for 2 min and then stirred magnetically at room temperature for 2 h, after which the acquired good dispersion PVA/CS@MWCNTs mixture was ready to fabricate film. An automatic film scraper was used to prepare PVA/CS@MWCNTs composite films. The stainless steel plate was selected as the substrate. The film scraping speed was 85 mm/s, and the film scraping thickness was set to 0.1 mm. After drying at 25 °C for 1 h and 80 °C for 12 h in a vacuum oven, composite films were removed from the substrate. The mass fraction (wt%) of filler MWCNTs was calculated by the following equation:

(1) $$\mathrm{wt}\%\;=\frac{M_{MWCNTs}}{M_{PVA}+M_{MWCNTs}}\times 100\%$$

**Figure 1:**
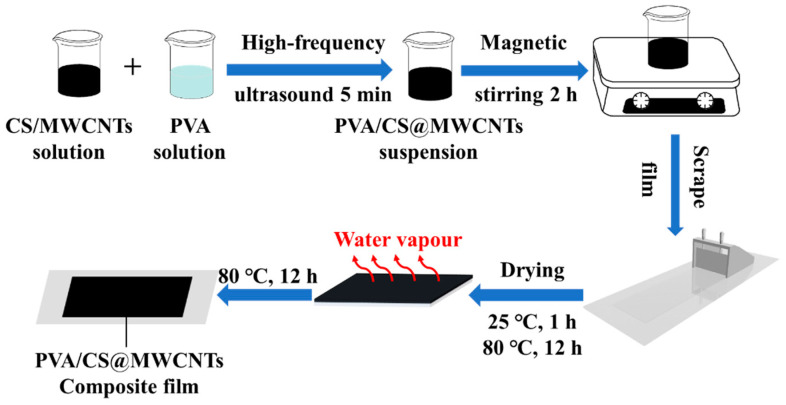
Schematic of the fabrication of sandwich-structured flexible PVA/CS@MWCNTs composite films.

Here *M_MWCNTs_* and *M_PVA_* represent the mass of MWCNTs and PVA, respectively. Then the mass fraction of MWCNTs in the composites was 1, 3, 5, and 7 wt%, respectively. For comparison, pure PVA film was prepared by the same method. The thickness of composite films was about 0.06~0.13 mm. The schematic diagram of the preparation process of PVA/CS@MWCNTs composite films is shown in Figure 1.

### 2.5. Characterization 

Scanning electron microscopy (SEM, JSM-6700F) and transmission electron microscope (TEM, JEOL-2100F, 100 KV) were used to observe the morphology and microstructures of films. XRD patterns of the prepared samples were determined by X-ray diffraction (XRD) of a D8-Advance Instrument (Bruker AXS) with Cu Kα radiation (λ = 1.5418 Å). The Fourier transform infrared (FTIR) spectrum was recorded on Nicolet iS 50, and attenuated total reflection (ATR) mode was carried out to test films in the range of 4000–500 cm^−1^. X-ray photoelectron spectroscopy (XPS) was employed to investigate the morphology of MWCNTs, CS and CS@ MWCNTs. Thermogravimetric analysis (TGA) was performed on a thermal analyzer system under N_2_ protection at a heating rate of 10 °C·min^−1^ (METTLER TOLEDO, TGA 2). κ = α·ρ·C_p_ was used to calculate the thermal conductivity of different films, in which α represents the thermal diffusivity, ρ represents the mass density, and C_p_ represents the heat capacity. The transient “laser flash” method (Nanoflash LFA 447) was used to measure the thermal diffusivity (α) of different samples. The calculation formula for density ρ is ρ = m/V, where m and V are the mass and volume of the test sample respectively. The differential scanning calorimeter (METTLER TOLEDO, DSC 3) with the sapphire method was carried out to measure the Cp of samples. More details of thermal tests have been described in detail in our previous work [33]. The thermal conductivity enhancement (κe) compared with pure PVA can be calculated as follow:

(2) $$\kappa e\left(\%\right)=\frac{\kappa -\kappa_{m}}{\kappa_{m}}\times 100\%$$

where κ represents the thermal conductivity of the composite films and $\kappa_{m}$ represents the thermal conductivity of the pure PVA. The films were cut into fixed geometry (diameters > 5.2 cm) for volume resistivity tests, which were measured by a ZC36 high insulation resistance measuring instrument. The mechanical performances of films were tested by a tensile tester at the rate of 15 mm/min at room temperature (Linkam, Redhill, UK, TST, 250 V). In order to make sure the test results were reliable, three specimens were tested for each sample.

## 3. Results and Discussions

### 3.1. Chitosan Coating on the Surface of Carbon Nanotubes

**Figure 2:**
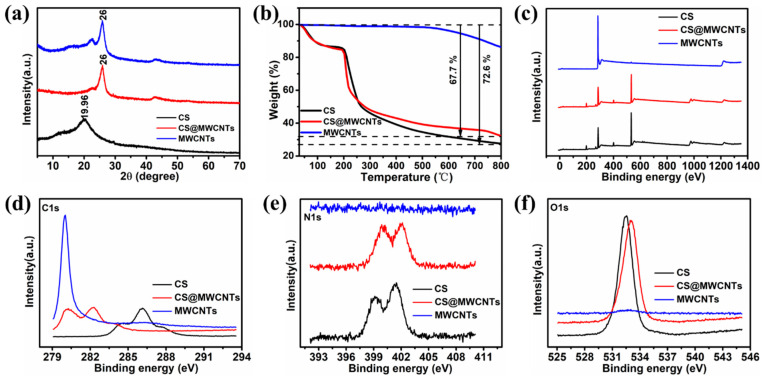
(**a**) XRD patterns of CS, MWCNTs, and CS@MWCNTs; (**b**) TGA curves in nitrogen atmosphere of CS, MWCNTs, and CS@MWCNTs; (**c**) XPS wide scan spectra of CS, MWCNTs, and CS@MWCNTs and corresponding high-resolution spectra of (**d**) C 1 s, (**e**) N 1 s, (**f**) O 1 s, respectively.

Fillers well dispersed in polymer matrix are very important to obtain ideal performance [34]. In order to disperse MWNTs well in the polymer matrix, CS was used to non-covalently modify MWNTs. A series of tests was utilized to verify the successful adsorption of chitosan on the surface of carbon nanotubes. Figure 2a exhibited the XRD patterns of CS, MWCNTs, and CS@MWCNTs. The characteristic peak of MWCNTs was obvious at 26°, which was ascribed to the (002) crystal plane diffraction of the hexagonal graphite structure, indicating the multiwalled nature of CNT [35]. Chitosan showed strong peaks at 19.96°, which corresponded to characteristic diffraction peaks of the crystal plane (040) [36]. In the XRD patterns of CS@MWCNTs, the characteristic peaks of CS and MWCNTs both appeared, confirming the existence of both CS and MWCNTs in this composite, MWCNTs, and CS only interact physically without chemical reaction. Meanwhile, CS crystallinity (calculated according to Debye Scherrer equation D = $\frac{0.89\lambda }{\beta Cos\theta }$) [37] decreased in CS/MWCNTs, indicating that CS chains were well distributed on the surface of MWCNTs [38].

The modification amounts of CS on MWCNTs surfaces were evaluated using TGA under a nitrogen atmosphere (Figure 2b). There was almost no weight loss for MWCNTs from 30 to 400 °C; with the increase in temperature, MWCNTs displayed slight weight losses of 13.8% at 800 °C. This is related to defects on the surface of carbon nanotubes, such as topological defects, heavy hybridization defects and incomplete bonding defects, which contribute to the dissociation of oxygen and lead to the formation of carbon-oxygen bonds. When heated to higher temperatures (800 °C), these oxygen-containing groups gradually disappeared. For CS and CS@MWCNTs, the weight loss under 200 °C was owing to the adsorption of water by physical desorption, and the weight reduction between 280 and 360 °C was due to the degradation and deacetylation of chitosan [39,40], at higher temperatures, additional weight loss occurred due to the further condensation of species on the carbonaceous surface [41]; When heated to 800 °C, the residual weight of CS@MWCNTs was about 27.4%, suggesting that the content of CS in CS@MWCNTs was about 58.8%. 

Figure 2c–f showed the XPS analysis of CS, MWCNTs, and CS@MWCNTs, which is a method for quantitative analysis of material surfaces. CS@MWCNTs showed characteristic peaks similar to CS in the spectral scanning XPS analysis, and the carbon spectrum displayed that the carbon peak intensity of CS@MWCNTs was between the spectral intensity of MWCNTs and CS. Because chitosan was coated on the surface of MWCNTs, the number of *sp*^2^ carbon atoms in the MWCNTs strongly attached to CS molecules increased, and the appearance of N and O peaks in CS@MWCNTs also indicated the presence of CS on the surface of MWCNTs.

**Figure 3:**
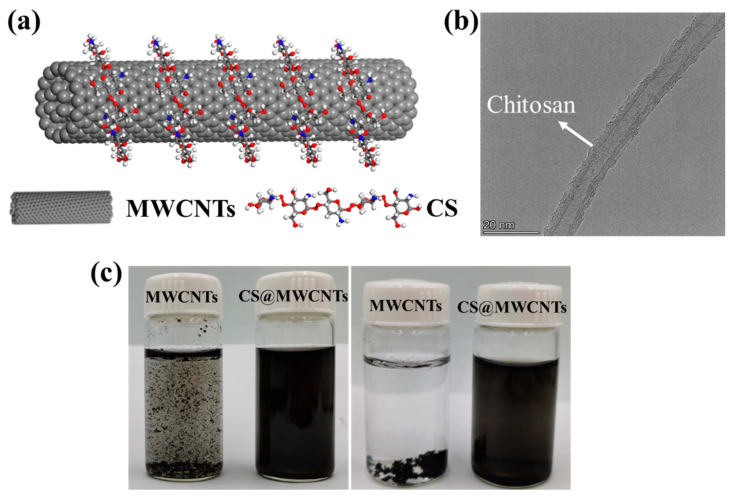
(**a**) Schematic diagram of CS-adsorbed on the surface of MWCNTs; (**b**) The TEM micrographs of CS-adsorbed on the surface of MWCNTs; (**c**) Photos of MWCNTs and CS@MWCNTs solubility in water one day on the left and 30 days on the right.

CS (chitosan) adsorbed on the surface of MWCNTs is shown in Figure 3a. The TEM result of chitosan adsorption on the surface of MWCNTs is also shown in Figure 3b. The comparison of dispersion of MWCNTs in aqueous solution before and after chitosan modification is shown in Figure 3c; this phenomenon suggested that CS significantly promoted the dispersion of MWCNTs. 

### 3.2. Dispersion and Interaction of CS@MWCNTs in PVA Matrix

**Figure 4:**
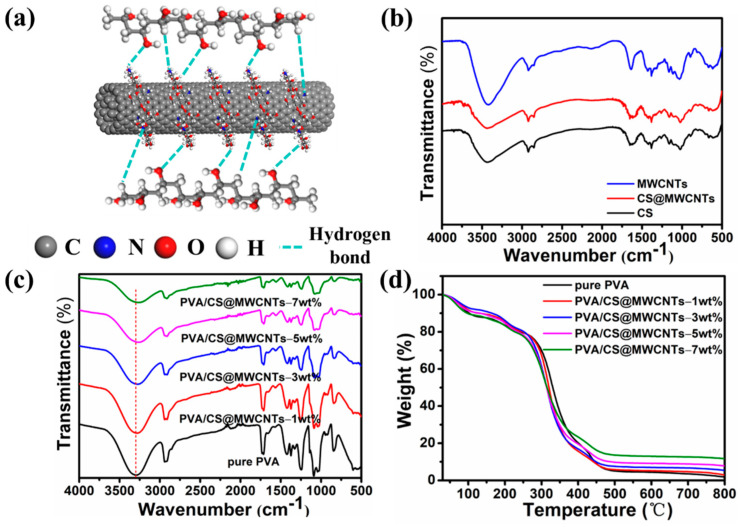
(**a**) Illustration of the interactions between PVA matrix and CS@MWCNTs by hydrogen bonds; (**b**) FTIR spectra of CS, MWCNTs and CS@MWCNTs; FTIR spectra (**c**) and TGA curves under nitrogen atmosphere (**d**) of pure PVA and PVA/CS@MWCNTs composite films containing 1 wt%, 3 wt%, 5 wt%, and 7 wt% MWCNTs.

Good interfacial interaction between fillers and polymer matrix can effectively reduce the interfacial thermal resistance and improve the thermal conductivity of PVA/CS@MWCNTs composite films [42]. CS has abundant amino and hydroxyl groups on its macromolecular chains; the interfacial interactions between CS@MWCNTs and PVA matrix are mainly through hydrogen bonds (as shown in Figure 4a,b), and the change of OH wavenumber in composites is related to hydrogen bond strength [43,44,45]. In Figure 4c, the OH peak at 3307 cm^−1^ of pure PVA was caused by the symmetric stretching vibration of hydroxyl groups on the PVA molecular chains [25]. Compared with pure PVA, the OH absorption peaks of PVA/CS@MWCNTs composite films shifted to a lower wave number, which may be related to the partial hydrogen bond dissociation between PVA molecular chains and the formation of the hydrogen bond between CS@MWCNTs and PVA matrix [46,47]. Those results all suggest that a good hydrogen bonding interface can be formed between fillers and the PVA matrix. Figure 4d depicted the weight loss trend of all composite films and pure PVA in the whole temperature range, which was similar. By contrast, the residual weight of the PVA/CS@MWCNTs composite films increased with the increase in filling amount, suggesting that the introduction of MWCNTs improved the thermal stability of the PVA/CS@MWCNTs composite films.

### 3.3. Microstructure of the Prepared PVA/CS@MWCNTs Composite Films

**Figure 5:**
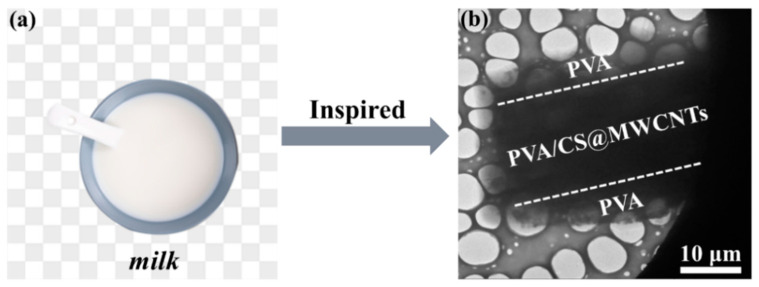
(**a**) Photographs of surface film formation of milk; (**b**) TEM fracture morphology of the PVA/CS@MWCNTs-5 wt% composite film.

Inspired by the surface film formation of milk, we designed and fabricated PVA/CS@MWCNTs films with a sandwich structure, adopting a self-construction strategy. The drying and forming process was divided into two stages, 25 °C for 1 h and then 80 °C for 12 h to provide delamination conditions (as shown in Figure 1). The TEM graphic of the fracture microstructures of the PVA/CS@MWCNTs composite film with 5 wt% is shown in Figure 5b; a sandwich structure can be obviously observed. The thinner and transparent layers are PVA and the darker middle layer is the PVA-CS@MWCNTs thermal conductivity layer. 

**Figure 6:**
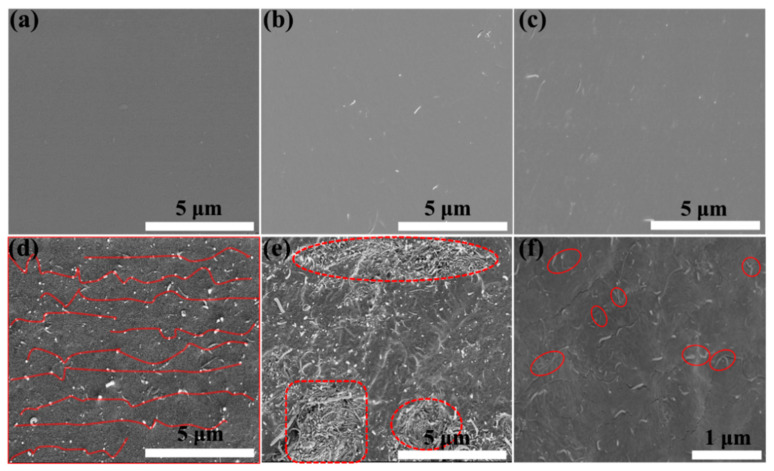
SEM cross-sectional micrograph of (**a**) pure PVA film; (**b**–**e**) PVA/CS@MWCNTs composite film with a loading of 1 wt%, 3 wt%, 5 wt%, and 7 wt% MWCNTs; SEM surface micrograph of PVA/CS@MWCNTs composite film with a 5 wt% MWCNTs loading.

The SEM cross-sectional micrograph of different films is shown in Figure 6a–e; the bright spots represented MWCNTs. Apparently, the cross-sectional micrograph of pure PVA (Figure 6a) was flat and smooth without any spots. However, as shown in Figure 6b–e, after MWCNTs were added to the PVA matrix, the smoothness of the PVA/CS@MWCNTs composite membranes decreased. At low filler loadings, the surface of the PVA/CS@MWCNTs composite film was relatively smooth with a few bright spots, and there was no heat conduction path in composite films. Thus, the thermal conductivity of the composite films was also relatively low. When the filler content increased to 5 wt%, the bright spots in the composite film increased and were evenly distributed, forming a good thermal conductivity path marked in the red line in Figure 6d, which could effectively improve the thermal conductivity of the composite film. When the MWCNTs content continuously increased to 7 wt%, a severe agglomeration of MWCNTs could be obviously observed in the composite film as displayed in Figure 6e, which was not conducive to improving the properties of the composite films. Therefore, the thermal conductivity of PVA/CS@MWCNTs-7 wt% composite film would decrease. In addition, an SEM surface micrograph of PVA/CS@MWCNTs-5 wt% composite film was also observed in Figure 6f; there were a small number of MWCNTs cross-linked nodes, which contributed to the heat transfer of phonons and improved the thermal conductivity of the composite film.

### 3.4. Thermal Conductivity Properties and Analysis of PVA/CS@MWCNTs Composite Films

**Figure 7:**
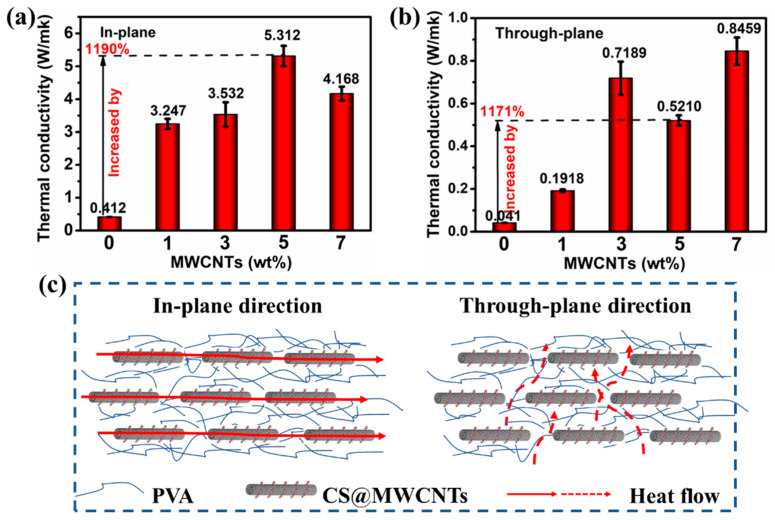
In-plane (**a**) and through-plane (**b**) thermal conductivity (ĸ) of pure PVA and PVA/CS@MWCNTs composite films varied with the loading of MWCNTs; (**c**) Schematic diagram of thermal conductivity of PVA/CS@MWCNTs composite films.

Compared with the pure PVA, the thermal conductivity of PVA/CS@MWCNTs composite films acquired great improvement with the increase in filler content, as shown in Figure 7a,b. When 5 wt% MWCNTs were added, the in-plane thermal conductivity reached a maximum of 5.312 W·m^−1^·K^−1^, which was 1190% higher than that of pure PVA (0.412 W·m^−1^·K^−1^). When more MWCNTs were added, the thermal conductivity decreased due to fillers agglomeration, as shown in the red circle in Figure 6e. The great enhancement of in-plane thermal conductivity was attributed to the horizontal orientation of the fillers in the matrix (Figure 7c), which formed a thermal conductivity path. This phenomenon is related to the composite film preparation process, during the process of scraping the film, the scraper exerted an external force on the horizontal direction, so that the fillers of MWCNTs tended to be arranged in the horizontal direction, which was beneficial to improve heat conduction along the horizontal direction. The in-plane thermal conductivity of our prepared PVA/CS@MWCNTs composite films was compared with the recently reported composite thermal conductivity films, and the results are summarized in Table 1.

In addition, the thermal conductivity of films in the in-plane direction is significantly higher than that in the through-plane direction (as shown in Figure 7b). There are two main reasons for the low through-plane thermal conductivity of the films; one is that the fillers were arranged along the horizontal direction under the shear force generated during the scraping process, and there are no effective heat conduction paths in the vertical direction as displayed in Figure 7c. The other is that the PVA distributed on both sides of the sandwich structure is also unfavorable to heat transfer in the vertical direction. This phenomenon can effectively attenuate the effect of anisotropic heat transfer on adjacent electronic components in thermal management [48].

### 3.5. Electrical Insulating Properties and Flexibility Demonstration of PVA/CS@MWCNTs Composite Films

**Figure 8:**
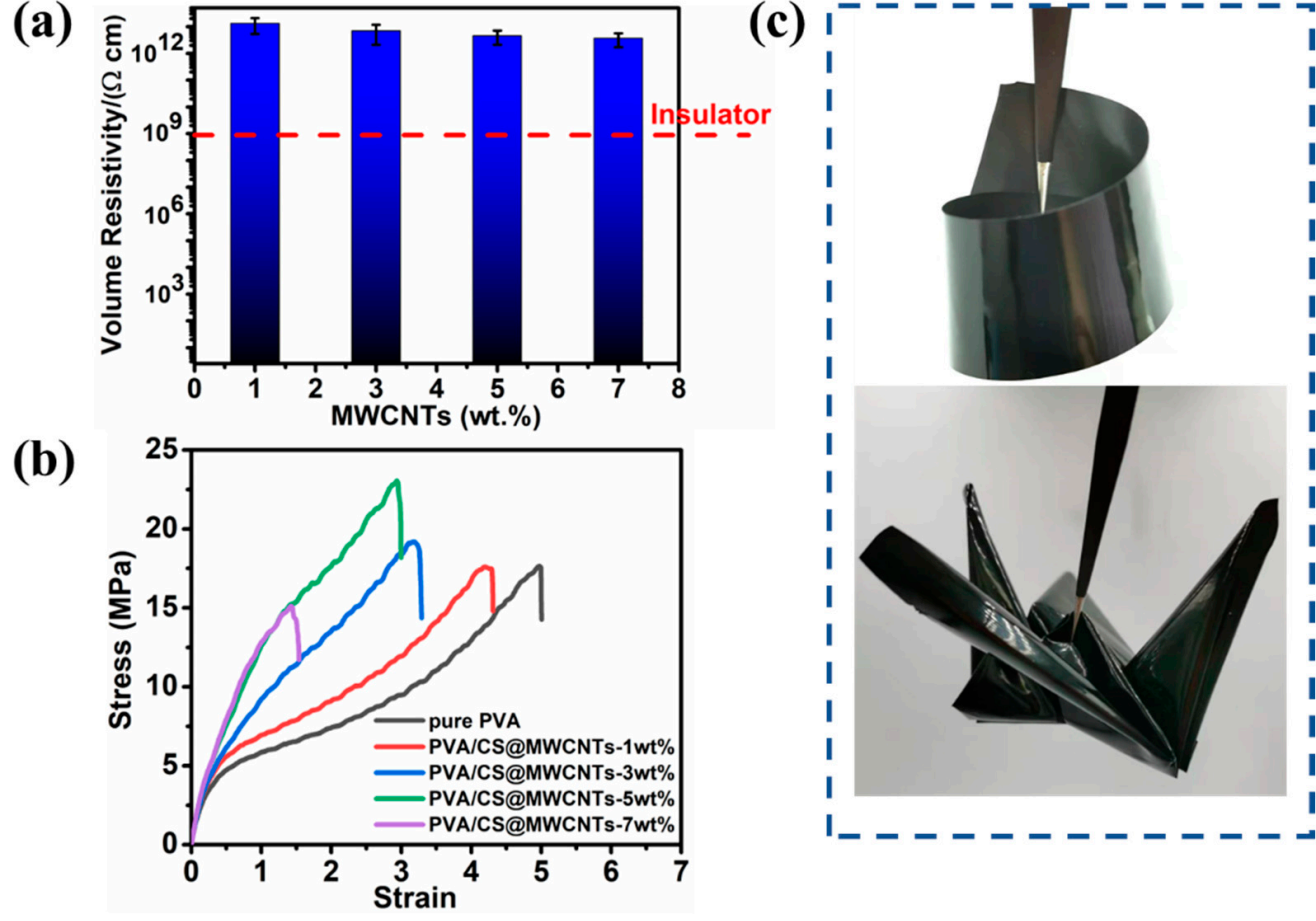
(**a**) Volume resistivity of PVA/CS@MWCNTs composite films varied with the loading of MWCNTs; (**b**) Mechanical property of pure PVA and PVA/CS@MWCNTs composite films with different content of MWCNTs; (**c**) PVA/CS@MWCNTs composite film with a content of 5 wt% MWCNTs folded into different shapes, showing excellent flexibility.

Considering the electrical insulation performance of thermal management materials required by electronic products, the volume resistivity of composite films was tested, and the results are shown in Figure 8a. Apparently, the volume resistivity of the composite films decreased slightly with the increase in filler content, but they are still highly insulating materials. Compared with pure PVA, the thermal conductivity of the PVA/CS@MWCNTs increased 1190% when the MWCNTs content was 5 wt%; however, its volume resistivity was 4.6 × 10^12^ Ω·cm. Those results indicated that the prepared composite films could keep the desirable electrical insulation property while increasing thermal conductivity. Such unique performances reported in this work are rare in the field of polymer matrix composites.

In addition to high thermal conductivity and good electrical insulation properties, the prepared PVA/CS@MWCNTs composite films also showed excellent flexibility. The mechanical properties of composite films were characterized, which is of great significance to the practical application of composite materials. In this work, tensile tests were employed to assess the mechanical properties as shown in Figure 8b. The mechanical properties of those composite films were significantly increased compared to the pure PVA. The addition of CS@MWCNTs could obviously improve the tensile strength of the PVA, which was mainly due to the strong hydrogen bond between PVA matrix and CS molecules. For the same reason, the value of elongation at break decreased with respect to confined and almost non-gliding molecules. The addition of CS@MWCNTs into the PVA matrix led to an obvious enhancement in tensile strength from 17.55 MPa of pure PVA to 22.97 MPa of PVA/CS@MWCNTs-5 wt% (Table 2), and the rate of enhancement reached up to 30.5%. After the PVA/CS@MWCNTs composite film containing 5 wt% MWCNTs was folded into different shapes (as shown in Figure 8c), no cracks and damage were found on the surface of the film. This phenomenon shows that PVA/CS@MWCNTs composite films loaded with 5 wt% MWCNTs still have good mechanical flexibility and have broad application prospects in flexible electronic devices.

## 4. Conclusions

In this study, we prepared PVA/CS@MWCNTs composite film with a sandwich structure inspired by the surface film formation of milk; the middle layer of PVA-CS@MWCNTs as a thermal conductivity layer is conducive to increasing the thermal conductivity of composite films, and the polymer distributed on both sides of the middle layer maintains electrical insulation. CS coating on the surface of MWCNTs can effectively improve the uniform dispersion of MWCNTs in the polymer matrix, and form a good interface bonding with the PVA matrix through hydrogen bonding, reducing the interfacial thermal resistance. In addition, the shear force generated during the scraping process could promote the orientation arrangement of MWCNTs in the in-plane direction. Thus, a good heat conduction path could be formed in the horizontal direction. The produced PVA/CS@MWCNTs-5 wt% film simultaneously showed superior in-plane thermal conductivity (5.312 W·m^−1^·K^−1^), good electrical insulation above 10^12^ Ω·cm (beyond electrical insulation of 10^9^ Ω·cm), excellent mechanical properties (tensile strength of 23.1 MPa) and outstanding flexibility. Our work has provided inspiration for the design of sandwich structure polymer composites, which have great application potential in the field of thermal management, especially in flexible electronic devices and electrical insulation.

## Figures and Tables

**Table 1:** Thermal conductivity of polymer composites with MWCNTs.

Filler	Filler Loading	Matrix	κ (W·m^−1^·K^−1^)	Volume Resistivity (Ω·cm)	Refs.
MWCNTs	19.3 vol%	PS-b-P4VP	0.73	-	[49]
MWCNTs	3.5 wt%	PP	0.87	-	[50]
MWCNTs	1 vol%	PA6	0.352	1.0 × 10^13^	[51]
MWCNTs	2 vol%	PPS/PE/EGMA	0.57	1.9 × 10^15^	[52]
MWCNTs	5 wt%	PVDF	0.83	1.2 × 10^13^	[53]
MWCNTs	7.4 wt%	NR	0.25	-	[54]
MWCNTs	35 wt%	NFCs	14.1	10^10^	[55]
MWCNTs	5 wt%	PVA	5.312	4.6 × 10^12^	This work

**Table 2:** Representative mechanical properties of the samples.

Samples	Tensile Strength (MPa)	Elongation at Break
Pure PVA	17.55 ± 0.62	5.14 ± 0.14
PVA/CS@MWCNTs-1 wt%	17.83 ± 1.50	4.26 ± 0.18
PVA/CS@MWCNTs-3 wt%	19.26 ± 0.31	3.21 ± 0.09
PVA/CS@MWCNTs-5 wt%	22.97 ± 0.14	2.70 ± 0.18
PVA/CS@MWCNTs-7 wt%	14.75 ± 0.83	1.56 ± 0.06
